# Proximal versus distal diuretics in congestive heart failure

**DOI:** 10.1093/ndt/gfae058

**Published:** 2024-02-29

**Authors:** Massimo Nardone, Vikas S Sridhar, Kevin Yau, Ayodele Odutayo, David Z I Cherney

**Affiliations:** University Health Network, Division of Nephrology, Department of Medicine, University of Toronto, Ontario, Canada; University Health Network, Division of Nephrology, Department of Medicine, University of Toronto, Ontario, Canada; University Health Network, Division of Nephrology, Department of Medicine, University of Toronto, Ontario, Canada; University Health Network, Division of Nephrology, Department of Medicine, University of Toronto, Ontario, Canada; University Health Network, Division of Nephrology, Department of Medicine, University of Toronto, Ontario, Canada

**Keywords:** carbonic anhydrase inhibitors, heart failure, mineralocorticoid receptor antagonists, sodium glucose cotransport 2 inhibitors, thiazide diuretics

## Abstract

Volume overload represents a hallmark clinical feature linked to the development and progression of heart failure (HF). Alleviating signs and symptoms of volume overload represents a foundational HF treatment target that is achieved using loop diuretics in the acute and chronic setting. Recent work has provided evidence to support guideline-directed medical therapies, such as sodium glucose cotransporter 2 (SGLT2) inhibitors and mineralocorticoid receptor (MR) antagonists, as important adjunct diuretics that may act synergistically when used with background loop diuretics in people with chronic HF. Furthermore, there is growing interest in understanding the role of SGLT2 inhibitors, carbonic anhydrase inhibitors, thiazide diuretics, and MR antagonists in treating volume overload in patients hospitalized for acute HF, particularly in the setting of loop diuretic resistance. Thus, the current review demonstrates that: (i) SGLT2 inhibitors and MR antagonists confer long-term cardioprotection in chronic HF patients but it is unclear whether natriuresis or diuresis represents the primary mechanisms for this benefit, (ii) SGLT2 inhibitors, carbonic anhydrase inhibitors, and thiazide diuretics increase natriuresis in the acute HF setting, but implications on long-term outcomes remain unclear and warrants further investigation, and (iii) a multi-nephron segment approach, using agents that act on distinct segments of the nephron, potentiate diuresis to alleviate signs and symptoms of volume overload in acute HF.

## INTRODUCTION

The retention of sodium and water and consequential fluid accumulation in the extracellular compartment is a cardinal feature of heart failure (HF), irrespective of the left ventricular ejection fraction [1, 2]. The expansion of the extracellular volume, manifesting as volume overload, represents a leading cause for HF hospitalization, with severity of volume overload associated with adverse HF outcomes [3, 4]. Alleviating volume expansion through the use of loop diuretics is a cornerstone of HF treatment [5–7]. However, achieving decongestion represents a longstanding challenge due to multiple physiological adaptations that favour the retention of sodium, with persistent fluid overload despite increasing diuretic dosing (i.e. diuretic resistance) representing a cardinal sign of worsening HF. Indeed, low urine output in response to loop diuretics or the need for repeated short-term intravenous administration of loop diuretics, are both associated with mortality in the acute HF setting [8, 9]. Furthermore, high diuretic dose requirements or dose escalation in the chronic HF setting are associated with hospitalization [10–12].

Accordingly, there is growing interest in understanding the diuretic and natriuretic properties of contemporary medical therapies such as sodium glucose cotransporter 2 (SGLT2) inhibitors, carbonic anhydrase (CA) inhibitors, thiazide diuretics, and mineralocorticoid receptor (MR) antagonists, and their roles in managing volume overload in HF, especially as adjuncts to loop diuretic therapy. The current review aims to summarize existing physiological and clinical evidence on the use of SGLT2 inhibitors, CA inhibitors, thiazide diuretics, and MR antagonists in managing HF and their role as adjunctive diuretic agents in the acute and chronic setting.

### Proximal tubular diuretics

**Figure 1:**
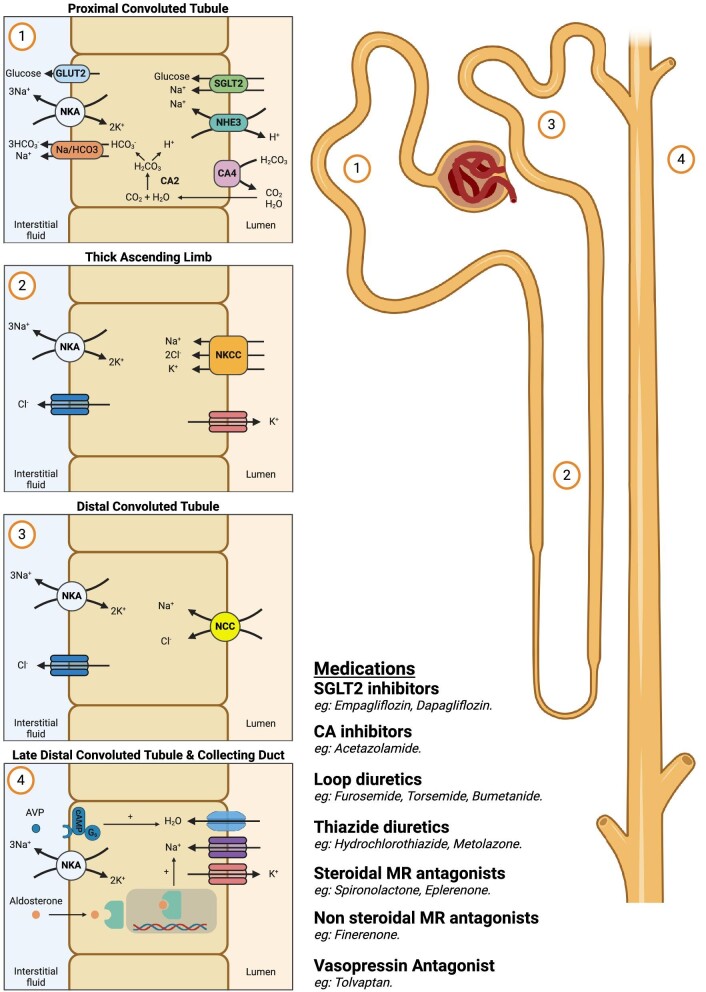
Schematic of the nephron with the site of action of adjunctive diuretic agents. SGLT2 inhibitors act on the SGLT2 and the NHE3 in the proximal tubule. CA inhibitors also act in the proximal convoluted tubule to prevent sodium and bicarbonate (HCO_3_) reabsorption through the NHE3 and Na-HCO_3_ cotransporters. Both SGLT2 inhibitors and CA inhibitors allows for increased distal delivery of sodium that may facilitate loop diuretic action. Loop diuretics act in the thick ascending limb by inhibiting the NKCC. Thiazide diuretics act on the distal convoluted tubule by inhibiting NCC and may be beneficial in the setting of distal tubular hypertrophy, a major mechanism of diuretic resistance. MR antagonists act in the late distal collecting tubule and collecting duct, by inhibiting the binding of aldosterone to the MR in the principal cells. This results in decreased sodium-potassium ATPase (NKA) and epithelial sodium channel activity at the basolateral and luminal membrane, respectively, resulting in increased natriuresis and potassium retention. Last, AVP can bind to the V_2_ receptor in the collecting duct to initiate secondary signalling and increased aquaporin permeability, leading to increased free water reabsorption.

Under normal physiological conditions, the proximal tubule is responsible for two-thirds of sodium reabsorption, principally mediated through the sodium-hydrogen exchanger isoform-3 (NHE3) and the sodium glucose cotransporter-2 (SGLT2) (Fig. 1) [1]. Neurohormonal activation, including increased plasma levels of norepinephrine, angiotensin II, and vasopressin, characterizes an early physiological manifestation contributing to sodium and bicarbonate (HCO_3_) retention in HF by upregulating SGLT2, Na-HCO_3_, and NHE3 activity in the proximal tubule [13]. Importantly, increased proximal tubular reabsorption leads to decreased downstream sodium delivery thereby influencing the effectiveness of loop diuretics.

### SGLT2 inhibitors

SGLT2 inhibitors are the most recent addition to guideline-directed medical therapy in HF patients with either reduced (HFrEF) or preserved (HFpEF) ejection fraction [14–19]. These medications have natriuretic and diuretic effects, as evaluated in numerous mechanistic and clinical studies (Table 1). Whether and to what extent the diuretic effects of SGLT2 inhibition contribute to improvements in HF outcomes remains a point of active debate.

**Table 1:** Summary of RCTs of diuretic agents in patients with HF.

Class	Clinical trial	Type	Cohort	Duration	Treatment	Outcomes
SGLT2 inhibitor	EMPA [26, 59]	Double-blind, placebo-controlled crossover RCT	HFrEF HFpEF	14 days	Empagliflozin 10 mg *q.d*. vs. Placebo	↑ FENa ↓ Total blood volume ↓ PV^b^ ↔ NT-proBNP ↓ Body weight
	RECEDE-CHF [31]	Double-blind, placebo-controlled crossover RCT	HFrEF	6 weeks	Empagliflozin 25 mg *q.d*. vs. Placebo	↑ Urine output ↔ FENa ↔ NT-proBNP
	Empire-HF [33, 38]	Double-blind, placebo-controlled RCT	HFrEF	12 weeks	Empagliflozin 10 mg *q.d*. vs. Placebo	↓ eECF ↓ ePV^a^ ↔ NT-proBNP ↔ PCWP
	MUSCAT-HF [34]	Open-label RCT	HFpEF	12 weeks	Luseogliflozin 2.5 mg *q.d* vs. Voglibose 0.2 mg *t.i.d*	↓ ePV^a^ ↔ BNP
	CANDLE [35]	Single-blind RCT	HFrEF HFpEF	24 weeks	Canagliflozin 100 mg *q.d* vs. Glimepiride 0.5 mg *q.d*	↓ eECF ↓ ePV^a^ ↔ NT-proBNP
	EMPA-RESPONSE-AHF [46, 47]	Double-blind, placebo-controlled RCT	AHF	96 hours	Empagliflozin 10 mg *q.d*. vs. Placebo	↑ Urine output ↔ Loop diuretic efficiency^c^ ↔ NT-proBNP ↔ FENa
	DAPARESIST [49]	Open-label, randomized, active-comparator, controlled RCT	AHF	96 hours	Dapagliflozin 10 mg *q.d*. vs. Metolazone 5–10 mg *q.d*.	↔ Body weight ↔ Loop diuretic efficiency^c^ ↔ Volume assessment score ↔ NT-proBNP
	EMPAG-HF [48]	Double-blind, placebo-controlled RCT	AHF	120 hours	Empagliflozin 25 mg *q.d*. vs. Placebo	↑ Urine output ↑ Loop diuretic efficiency^c^ ↓ NT-proBNP ↔ Body weight
	EMPULSE [50]	Double-blind, placebo-controlled RCT	AHF	30 days	Empagliflozin 10 mg *q.d*. vs. Placebo	↑ Loop diuretic efficiency^c^ ↓ NT-proBNP ↔ Rehospitalization (30 days)
CA inhibitor	DIURESIS-CHF [53]	Single-blind RCT	AHF	72 hours	Acetazolamide 500 mg *q.d*. and Bumetanide 2 mg *b.i.d* vs. Bumetanide 2 mg *b.i.d*	↔ Urinary Na volume ↔ NT-proBNP ↑ Loop diuretic efficiency^c^
	ADVOR [54, 55]	Double-blind, placebo-controlled RCT	AHF	72 hours	Acetazolamide 500 mg *q.d*. vs. Placebo	↑ Urine output ↑ Urine sodium ↑ Decongestion^d^ ↔ Rehospitalization (30 days)
Thiazide diuretic	3T [60]	Double-blinded, double-dummy RCT	AHF	48 hours	Metolazone 5 mg *b.i.d* vs. Hydrochlorothiazide 500 mg *b.i.d* vs. Tolvaptan 30 mg *q.d*.	↔ Body weight ↔ Urine output ↔ Patient congestion score ↑ FENa ↔ Diuretic efficiency
	CLOROTIC [69]	Double-blind, placebo-controlled RCT	AHF	96 hours	Hydrochlorothiazide 500 mg *b.i.d* vs. Placebo	↓ Body weight ↔ Dyspnoea ↑ Diuretic efficiency ↔ Rehospitalization (30 days)
MR antagonist	RALES [84]	Double-blind, placebo-controlled RCT	HFrEF	12 weeks	Spironolactone 25 mg *q.d*. vs. Placebo	↓ BNP ↓ Mortality & hospitalizations
	ARTS [73]	Double-blind, active-comparator, and placebo-controlled RCT	HFrEF	2 weeks	Finerenone 2.5–10 mg *q.d*. vs. Spironolactone 25–50 mg *q.d*. vs. Placebo	↓ BNP
	ARTS-HF [80]	Double-blind, active-comparator RCT	AHF	90 days	Finerenone 2.5–20 mg *q.d*. vs. Eplerenone 25–50 mg *q.d*.	↔ NT-proBNP
	ATHENA-HF [89]	Double-blind, placebo-controlled RCT	AHF	96 hours	Spironolactone 100 mg *q.d*. vs. Placebo	↔ NT-proBNP ↔ Congestion scores ↔ Urine output ↔ Body mass

ECF: extracellular fluid volume; PCWP: pulmonary capillary wedge pressure; PV: plasma volume. ^a^Plasma volume estimated using the Strauss formula. ^b^Plasma volume measured using indicator dilution with I-131 albumin. ^c^Loop diuretic efficacy was defined as a change in body weight normalized to loop diuretic dose. ^d^Successful decongestion was defined as the absence of signs of volume overload based on (i) no more than trace oedema, (ii) no residual pleural effusion, and (iii) no residual ascites.

Within the proximal tubule, SGLT2 is normally responsible for ∼5% of total sodium reabsorption in the kidneys [20]. SGLT2 inhibition reduces proximal tubular sodium and glucose reabsorption to promote natriuresis, glucosuria, and accompanying osmotic diuresis [21]. These medications also induce concurrent NHE3 inhibition [22], which further drives proximal tubular natriuresis and increased sodium delivery to the downstream macula densa. This is hypothesized to restore tubuloglomerular feedback leading to reductions in intraglomerular pressure and glomerular filtration rate, clinically manifesting as an acute and reversible decline in eGFR [23]. Inhibition of proximal tubular glucose and sodium reabsorption is also thought to have favourable effects on renal energetics, with subsequent effects on hypoxia, inflammation, and fibrosis [24, 25].

#### Natriuresis and diuresis in chronic HF

Acute SGLT2 inhibition causes an initial increase in proximal tubular natriuresis and parallel increase in total natriuresis, measured using fractional sodium and lithium excretion [26, 27]. This acute natriuretic response is further accentuated during concurrent SGLT2 inhibitor and loop diuretic administration, suggesting a synergistic response probably driven by upstream SGLT2 inhibition causing greater sodium delivery to the downstream loop of Henle [26]. While the SGLT2 inhibitor-mediated increase in total natriuresis was evident following 14 days of treatment [26], counterregulatory adaptations to tubular sodium handling can promote tachyphylaxis to total natriuresis [27]. For instance, proteomic analyses have found that empagliflozin can increase circulating CA and uromodulin, which can cause counterregulatory increases sodium reabsorption in the proximal tubule and loop of Henle, respectively [28]. SGLT2 inhibition can also activate aldosterone, due to intravascular volume contraction, further promoting sodium reabsorption in the distal tubule, while osmotic diuresis associated with SGLT2 inhibitor-mediated glucosuria can increase vasopressin to promote fluid retention [29, 30]. Perhaps as a result of these physiological changes, attenuated natriuresis was observed at 14 days compared to 1 day of SGLT2 inhibitor treatment, despite remaining elevated compared to placebo at 14 days [27]. Furthermore, urinary sodium excretion or fractional excretion of sodium were not different following longer-term (4–6 weeks) treatment with SGLT2 inhibitors compared to placebo control [31, 32]. Collectively, these findings suggest that counterregulatory adaptations to tubular sodium handling may offset the diuretic effect of SGLT2 inhibition in chronic HF patients.

The effects of SGLT2 inhibition on reducing plasma volume [26, 33–35] and extracellular volume also appear modest [33, 36, 37]. The EMPIRE-HF trial examined the change in extracellular volume using EDTA in HF patients randomized to empagliflozin versus placebo for 12 weeks [33]. Empagliflozin reduced estimated extracellular volume compared with placebo by a modest 0.12 l. Other studies also examined changes in extracellular volume with SGLT2 inhibition, often using bioimpedance spectroscopy, and demonstrated reductions ranging from 0.5 to 1 l that tended to attenuate with time [36]. These results are consistent with the tubular effects of SGLT2 inhibition discussed before. Furthermore, mechanistic studies have consistently demonstrated no effect of SGLT2 inhibition on N-terminal pro-B-type natriuretic peptide (NT-proBNP) following shorter-term (2–24 weeks) treatment [26, 31, 33–35, 38]. This finding aligns with the inconsistent reductions in surrogates for cardiac filling pressures following 12 weeks of SGLT2 inhibitor treatment [38, 39].

Across several dedicated HF outcome trials, SGLT2 inhibition consistently improved composite outcomes that included HF hospitalization. Mediation analyses have identified that haemoconcentration, reflected by a rise in haematocrit, represents an important contributor to improved HF outcomes [40]. However, it is unclear whether haemoconcentration occurs secondary to plasma volume contraction or erythropoiesis [41]. Furthermore, statistical significance in the composite HF outcomes was achieved by 4 weeks of therapy, in contrast to kidney outcome trials [42, 43]. This relatively rapid cardioprotective effect appears to coincide with the natriuresis and diuresis responses augmented early following treatment initiation, implying that acute diuresis may contribute to early cardioprotection. By contrast, other secondary analyses of HF outcome trials provide stronger evidence to support the notion that non-diuretic mechanisms largely contribute to the observed long-term cardioprotection. Secondary analysis of the EMPEROR-Reduced trial examined a sub-group of patients with volume overload in the 4 weeks before randomization [44]. These participants were more likely to be on higher doses of loop diuretic and experience a HF event. However, there was no heterogeneity in the benefit of SGLT2 inhibition among participants with and without volume overload, arguing against natriuresis and diuresis being the primary mediators of the effects of empagliflozin. In addition, secondary analysis of DAPA-HF found that dapagliflozin did not alter furosemide dosing over the course of study [45], yet secondary analyses of DELIVER and EMPEROR-Preserved found that dapagliflozin and empagliflozin were associated with greater likelihood for loop diuretic de-escalation, and lower likelihood for loop diuretic intensification or initiation [11, 12]. Regardless, improved HF outcomes with SGLT2 inhibitors were consistently independent of background diuretic dose or severity of volume overload [11, 12, 45]. Last, EMPEROR-Reduced found that empagliflozin improved HF outcomes irrespective of the baseline NT-proBNP [4]. Taken together, these results do not appear to argue in favour of overwhelming diuretic effects with SGLT2 inhibition as the primary mechanism responsible for improvements in HF outcomes, but rather potential improvements in cardiac function and maintenance of kidney function that ultimately improve sodium balance and volume status.

#### Natriuresis and diuresis in acute HF

The efficacy of SGLT2 inhibitors on alleviating volume overload in the setting of acute HF have been investigated in several randomized control trials [46–50]. In the EMPA-RESPONSE-AHF trial, treatment with empagliflozin did not alter diuretic efficiency, defined as the change in body weight relative to loop diuretic dose, or NT-proBNP compared to placebo [46]. Furthermore, the increased urine output during treatment was mediated by glucosuria rather than natriuresis [47]. In the EMPAG-HF trial, higher empagliflozin dosage and earlier initiation following hospitalization resulted in increased urine output and loop diuretic efficiency, while decreasing NT-proBNP [48]. In addition, in patients with evidence of diuretic resistance, dapagliflozin demonstrated similar decongestive responsiveness compared to metolazone at 96 hours following treatment [49]. Finally, the EMPULSE trial demonstrated that early inpatient initiation of empagliflozin reduced the hierarchical composite outcome of all-cause mortality, HF events, or symptoms at 90 days versus placebo [50], while also improving diuretic efficiency and causing larger reductions in NT-proBNP within 30 days. These data collectively support an acute diuretic effect with SGLT2 inhibition contributing to clinical benefit.

#### Summary

**Figure 2:**
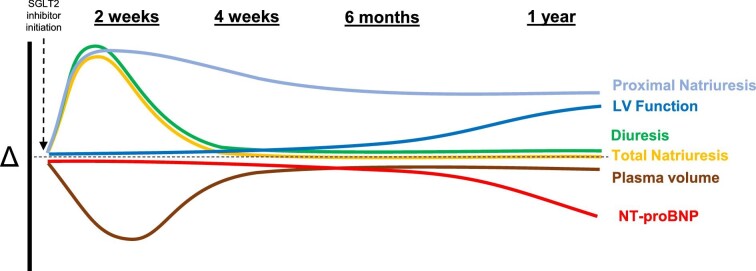
Proposed conceptual model illustrating the proposed physiological time-course following initiation of SGLT2 inhibitor treatment.

There is clear consensus that SGLT2 inhibition offers clinically meaningful benefits in the setting of HF. However, it is unclear to what extent, if any, these benefits are mediated by the diuretic effects of SGLT2 inhibitors. Natriuresis and diuresis appear to characterize early effects of SGLT2 inhibition that subsequently attenuates with time, suggesting activation of alternate pathways that confer clinical benefit (Fig. 2). It has also been proposed that a persistent effect of SGLT2 inhibition on proximal tubular natriuresis resets volume homeostasis which may allow individuals to better manage episodes of volume expansion, preventing exacerbations of HF.

### Carbonic anhydrase inhibitors

CA is a membrane-bound and luminal enzyme located in the proximal convoluted tubule, responsible for sodium and HCO_3_ reabsorption. Under normal physiological conditions, the breakdown and subsequent reabsorption of HCO_3_ into proximal tubule facilitates sodium reabsorption through the NHE3 and Na-HCO_3_ cotransporters, located on the apical and basolateral membrane, respectively (Fig. 1). Thus, acetazolamide, a CA inhibitor, prevents proximal tubular HCO_3_ reabsorption, subsequently limiting sodium reabsorption and resulting in natriuresis.

#### Natriuresis and diuresis in chronic HF

The natriuretic properties of acetazolamide were initially investigated for chronic HF treatment in the mid-1950s [51, 52]. However, the rapid diuretic resistance resulting from chronic treatment and the simultaneous emergence of loop diuretics precluded future use. As a result, CA inhibitors are not recommended for the treatment of chronic HF [5, 7].

#### Natriuresis and diuresis in acute HF

Acetazolamide has been investigated as an adjunct to loop diuretics in the setting of acute HF (Table 1). Acetazolamide was first explored in the DIURESIS-CHF trial, which enrolled acute HF patients presenting with evidence of volume overload and clinical suspicion for loop diuretic resistance [53]. Treatment with the combination of acetazolamide and loop diuretics versus high-dose loop diuretic monotherapy had no effect on the primary endpoint of 24-hour urinary sodium excretion, or the secondary outcomes of NT-proBNP [53]. However, combination therapy was associated with improvements in loop diuretic efficiency, defined in this study as natriuresis relative to loop diuretic dose, and a nominal improvement in time to hospital readmission.

The ADVOR trial was a multicentre randomized control trial of acute HF patients treated with intravenous acetazolamide or placebo in addition to standardized intravenous diuretic therapy [54]. The primary outcome of ‘successful decongestion’ was defined as the absence of residual congestion after 72 hours of treatment, with congestion graded on the basis of peripheral oedema, pleural effusion, or ascites. Successful decongestion occurred in 42% and 31% receiving acetazolamide and placebo, respectively, despite only a modest increase in diuresis based on urine output at 48 hours. Acetazolamide also increased total natriuresis, based on urinary sodium excretion [55]. While the ADVOR trial found that acetazolamide had no impact on the secondary outcome of death from any cause or HF rehospitalization at 3 months [54], greater natriuretic responsiveness was associated with favourable outcomes [55].

In secondary analyses of the ADVOR trial, patients with higher baseline serum HCO_3_ had a more pronounced reduction in congestion [56]. Higher serum HCO_3_ may reflect upregulation of HCO_3_ reabsorption in the proximal tubule secondary to sympathetic and neurohormonal overactivation [13]. Loop diuretic use is also associated with a higher incidence of metabolic alkalosis, which can further drive neurohormonal overactivation and attenuate diuretic effectiveness [57]. Patients undergoing diuresis with loop diuretics frequently develop elevated HCO_3_ related to chloride depletion, which thereby impairs HCO_3_ elimination through Pendrin, a chloride-HCO_3_ exchanger found in type-B intercalated cells. In the ADVOR trial, elevated baseline HCO_3_ levels were associated with higher doses of loop diuretics, higher serum sodium levels, and more pronounced peripheral oedema. These findings provide evidence to support the notion that acetazolamide may exert benefit by targeting excessive neurohormonal activation, to mitigate development of metabolic alkalosis and subsequent diuretic resistance. Interestingly, the decongestive effects of acetazolamide were consistent across the spectrum of left ventricular ejection fraction, despite established therapies targeting neurohormonal overactivation having proven benefit only in HFrEF [58].

It is important to consider that the ADVOR trial was conducted prior to the routine use of SGLT2 inhibitors for chronic HF, with prior treatment with an SGLT2 inhibitor representing an exclusion criterion. Thus, extrapolation to the current era in which SGLT2 inhibitors are indicated for HFrEF and HFpEF patients is uncertain given that both achieve diuresis and natriuresis via proximal tubular natriuresis including NHE3 inhibition effects. Further, those with advanced chronic kidney disease (eGFR <20 mL/min/1.73 m^2^) were excluded from ADVOR, while individuals treated with an intravenous loop diuretic dose of >80 mg furosemide equivalent during the index hospitalization did not proceed to randomization, suggesting those with severe diuretic resistance may have been excluded. Thus, future trials will be required to conclusively determine treatment efficacy in these high-risk patient cohorts.

#### Summary

Acetazolamide can be considered as an adjunctive treatment to loop diuretics in admitted patients with acute HF. Use of acetazolamide in HF patients in the chronic setting, or acute setting in high-risk patients, such as those with advanced chronic kidney disease or severe diuretic resistance, require further prospective work.

### Distal tubular diuretics

The distal convoluted tubule accounts for <10% of sodium reabsorption through the sodium-chloride cotransporter (NCC) (Fig. 1) [1,2]. However, distal sodium reabsorption can be altered by a variety of factors such as neurohormonal activation, tubular flow rate, aldosterone, and vasopressin, each of which can impact either the pathological development or therapeutic treatment of HF. Importantly, in the setting of chronic diuretic use or chronic HF, hypertrophy of the distal tubule increases distal tubular sodium reabsorption, contributing to sodium retention and is a major mechanism of diuretic resistance [59].

### Thiazide diuretics

Thiazide diuretics have conventionally been considered a first line adjunct to augment diuresis, targeting the maladaptive distal tubular hypertrophy, by decreasing sodium reabsorption in the distal convoluted tubule through inhibition of the Na-Cl cotransporter (Fig. 1). Thiazide diuretics include metolazone, which has a long duration of action and is effective in those with advanced chronic kidney disease, hydrochlorothiazide, chlorothiazide, and chlorthalidone. Both metolazone and hydrochlorothiazide cause similar acute changes in body weight, urine output, diuretic efficiency, and patient congestion scores, suggesting that neither have established superiority [60]. However, in the acute HF setting, metolazone treatment was associated with increased mortality compared to high-dose loop diuretic treatment in propensity analyses, although this may be attributed to confounding by indication [61]. Other thiazide diuretics, such as intravenous chlorothiazide and oral chlorthalidone, have also been used to promote diuresis to mitigate volume overload in acute HF; although the only available evidence supporting this practice is observational in nature [62, 63], and randomized controlled trial data is lacking.

#### Natriuresis and diuresis in chronic HF

Thiazide diuretics are most frequently utilized concurrently to loop diuretics for treating chronic HF patients in clinical practice, particularly in the setting of loop diuretic resistance. Indeed, loop diuretics and thiazide diuretics can act synergistically to help achieve adequate diuresis [64]. This synergistic response, mediated by distinct tubular mechanisms of action for sequential nephron blockade, forms the physiological rationale for combined loop and thiazide diuretics in consensus statements [5]. While observational studies support the addition of thiazide diuretics, such as metolazone, to improve weight loss and decongestion in patients with chronic HF [65], there is a lack of randomized controlled trial evidence in this setting.

#### Natriuresis and diuresis in acute HF

Several earlier trials have shown that the addition of a thiazide diuretic can increase diuresis and natriuresis in acute HF patients with profound loop diuretic resistance [66–68]. For instance, the 3T trial that randomized 60 patients with acute HF to metolazone, intravenous chlorothiazide, or tolvaptan, found that all strategies augmented urine output and weight loss [60]. The CLOROTIC trial provides new evidence for targeting the distal tubule with thiazide diuretics [69]. Patients with acute HF treated with background intravenous loop diuretics were assigned to either oral hydrochlorothiazide or placebo for 72 hours. Patients treated with hydrochlorothiazide observed larger reductions in body weight, although no difference in the co-primary endpoint of dyspnoea was observed. Further, the reductions in body weight did not persist at discharge. Hydrochlorothiazide also augmented urine output and increased natriuresis at 96 hours. No difference in serious adverse event risk was noted, although hypokalaemia occurred in 41% and 16% of patients prescribed hydrochlorothiazide and placebo, respectively. In addition, increases in serum creatinine were more common with hydrochlorothiazide, although this may be reflective of achievement of diuretic effect. In summary, the CLOROTIC study provides evidence that thiazide may provide a modest beneficial effect on natriuresis and diuresis in acute HF.

#### Summary

While thiazide diuretics are routinely used as adjunctive diuretics in chronic HF and recommended in clinical guidelines, there is a lack of randomized controlled data supporting beneficial impact on long-term outcomes. In addition, the CLOROTIC trial provides new evidence that hydrochlorothiazide may be used in the setting of acute HF to achieve decongestion.

### Mineralocorticoid receptor antagonists

There is longstanding evidence that aldosterone contributes to the development of volume overload [70]. MR antagonists inhibit binding of aldosterone to the MR in the principal cells of the distal nephron [71]. This causes downstream suppression of genes encoding for the Na-K ATPase at the basolateral membrane and the epithelial sodium channel at the luminal membrane, resulting in increased natriuresis and potassium retention (Fig. 1). First generation MR antagonists, such as spironolactone and eplerenone, were initially developed as steroidal hormones to directly mimic aldosterone binding, with consequential secondary off-target sex steroid-related side effects. Nonsteroidal MR antagonists, such as finerenone, were subsequently developed as highly selective and potent alternatives that similarly inhibit aldosterone binding in the principal cell, without sex steroid-related side effects [72]. The incidence of hyperkalaemia associated with finerenone was also lower than that of steroidal MR antagonists [73]. Further, finerenone also causes an initial eGFR dip [74] analogous to SGLT2 inhibitor treatment described above [23]. However, the mechanisms responsible for the eGFR dip are probably not a reflection of natriuresis, as observed with SGLT2 inhibition, but rather on the basis of vascular effects. Further detail on the physiological differences between spironolactone, eplerenone, and finerenone in the setting of cardiorenal disease can be found elsewhere [75].

In the pivotal RALES and EMPHASIS-HF trials, spironolactone and eplerenone reduced the incidence of all-cause mortality and hospitalization in HFrEF patients [76, 77], providing unequivocal evidence to support the inclusion of steroidal MR antagonists in guideline-directed medical therapy [5]. By contrast, in the TOPCAT trial, spironolactone did not reduce the incidence of all-cause death, cardiac arrest, or HF hospitalization in HFpEF patients [78], although the absence of a statistically significant effect on the primary outcome has been attributed to heterogeneity by region of enrolment [79]. Last, finerenone demonstrated non-inferiority in exploratory outcome analyses compared to eplerenone in HFrEF patients [80]. Forthcoming randomized clinical trials will further investigate finerenone in HFrEF and HFpEF (FINEARTS-HF: NCT04435626, CONFIRMATION-HF: NCT06024746, REDEFINE-HF: NCT06008197, FINALITY-HF: NCT06033950).

#### Natriuresis and diuresis in chronic HF

The use of spironolactone to elicit increases in natriuresis have been well-described [81]. Short-term spironolactone treatment increased urinary sodium excretion, resulting in decreases in net sodium balance, body weight, and neurohormonal activation in HF patients abstaining from diuretic use [82]. Furthermore, in severe HF patients with diuretic resistance, evident from persistent congestion despite maximal tolerable doses of loop diuretics and ACE inhibition, the addition of spironolactone further increased sodium excretion and decreased body weight [83], collectively supporting diuretic and natriuretic properties of spironolactone. MR antagonists also cause reductions in natriuretic hormones (Table 1). A secondary analysis of the RALES trial demonstrated that spironolactone decreased BNP following 3 months of treatment [84]. Further, both eplerenone and finerenone elicited comparable reductions in NT-proBNP following 3 months in HFrEF patients [80], while spironolactone decreased NT-proBNP following 9 months in HFpEF patients [85].

Synonymous to SGLT2 inhibitors, the rapid time to statistical benefit (20 days) in the composite endpoints in HFrEF patients prescribed MR antagonists [86] suggests that the mechanisms responsible for cardioprotection are promptly activated following treatment initiation. While the fast-acting diuretic effects may intuitively represent an important mediator, it is important to recognize that MR antagonists also possess anti-hypertrophic, anti-inflammatory, and anti-fibrotic effects that largely mediate the cardiovascular benefits noted in clinical trials. Importantly, there is evidence to support that these mechanisms represent the primary actions responsible for long-term cardiovascular benefit of MR antagonists, as opposed to the natriuretic and diuretic properties. For example, in a secondary analysis of the TOPCAT trial, patients treated with spironolactone required lower doses of furosemide and thiazide diuretics compared to placebo [87]. However, reductions in HF hospitalization were not solely explained by indices of diuresis, suggesting that non-diuretic mechanisms were responsible for mediating the observed clinical benefit [87]. Furthermore, spironolactone elicited improvements in mortality most clearly observed in patients with the lowest NT-proBNP [88]. Therefore, steroidal MR antagonists may have natriuretic and diuretic effects but the contribution of these changes to the overall cardiovascular benefit noted in dedicated clinical trials is unclear.

#### Natriuresis and diuresis in acute HF

Dedicated clinical trials have sought to define the role of MR antagonists in the management of acute HF (Table 1). The ATHENA-HF trial examined the efficacy of high- and low-dose spironolactone for 96 hours versus placebo on the primary outcome of change in NT-proBNP levels [89]. Of note, high-dose spironolactone had no significant effect on NT-proBNP compared to placebo or low-dose spironolactone. It also had no effect on urine output, weight change, loop diuretic dose, or patient reported outcomes [89]. The lack of benefit with short-term MR antagonism in acute HF may related to the prolonged onset of action of spironolactone. Therefore, any decongestive effects may not have been appreciated during the short follow-up of ATHENA-HF [90].

#### Summary

MR antagonists have natriuretic and diuretic properties, which may be potentiated when combined with proximal acting diuretics. The extent to which these natriuretic and diuretic properties underlie the long-term cardioprotective effects remains unclear. Nonetheless, given the clear net benefit of MR antagonists, their use is indicated in people with chronic HFrEF, and forthcoming studies of nonsteroidal MR antagonists will further inform their use in HFpEF.

### Vasopressin inhibitors

Arginine vasopressin (AVP) plays an integral role in free water regulation at the level of the distal tubule and collecting duct. AVP stimulates V_2_ receptors, initiating secondary signalling to increase aquaporin channel permeability, facilitating the reabsorption of free water to ultimately cause fluid retention and hyponatraemia (Fig. 1). From a physiological standpoint, AVP, or surrogate markers such as copeptin, are elevated in HF patients [91], and elevated copeptin is associated with mortality in acute HF patients [92].

#### Natriuresis and diuresis in chronic HF

In chronic HF patients on stable loop diuretic treatment without evidence for volume overload, tolvaptan initiation resulted in a prompt increase in weight loss and reduction in oedema [93]. However, there is no data to support the use of these agents in the setting of chronic HF [5, 7].

#### Natriuresis and diuresis in acute HF

There is consistent evidence across multiple randomized controlled trials that the vasopressin receptor antagonist, tolvaptan, has no effect on symptoms of congestion or hard clinical outcomes in acute HF patients. The ACTIV trial demonstrated that tolvaptan acutely increased weight loss and urine volume compared to placebo, but had no impact on signs or symptoms of volume overload at hospital discharge or worsening HF at 60 days post-discharge [94]. Importantly, the EVEREST trial subsequently demonstrated that all-cause mortality or cardiovascular death was similar in acute HF patients prescribed tolvaptan and placebo [95]. Last, the TACTICS-HF and SECRET trials found no difference in the proportion of patients who showed clinical improvement in volume overload following initiation of tolvaptan [96, 97]. This may reflect that tolvaptan results in aquaresis (free water excretion) but does not result in natriuresis and therefore may not address sodium retention as the underlying pathophysiologic process driving HF. Nonetheless, tolvaptan is efficacious for treating hyponatraemia [98], which may be considered for severe hyponatraemia in the setting of acute HF.

#### Summary

These data provide convincing evidence that tolvaptan can lead to greater reductions in body weight and increase in urine volume during short-term usage. However, these changes do not translate into improvements in signs and symptoms of volume overload, and longer-term outcomes of mortality or cardiovascular death in acute HF patients.

### A multi-nephron segment targeting approach to reduce volume overload

#### Chronic HF

Current ESC guidelines recommend four classes of medications (beta blockers, RAAS blockade, MR antagonists, and SGLT2 inhibitors) for HFrEF that, when used simultaneously, result in the greatest relative risk reduction in cardiovascular outcomes and hospitalization [99, 100]. Conventional treatment strategies involve initiating treatment sequentially at low dosages, with careful monitoring prior to either up-titration or implementation of additional therapies. However, recent emergence of simultaneous initiation and rapid up-titration of multiple HF therapies is gaining attraction as a superior approach towards improving HF outcomes [101, 102]. The STRONG-HF trial demonstrated that, compared to usual care, high-intensity treatment strategies employing rapid implementation and up-titration of guideline-directed therapies, resulted in greater reductions in congestion and incidence of hospital readmission following discharge [103]. However, reluctance towards adopting this approach arises from clinician concern of the potential risk of adverse effects including hyperkalaemia or medication intolerance, which require close patient monitoring. While loop diuretics, thiazide diuretics, and potassium binders may reduce hyperkalaemia, the kaliuretic effects of SGLT2 inhibitors have been recently shown reduce the incidence of hyperkalaemia associated with RAAS blockade or MR antagonists usage, and thus may also help to facilitate prescription of goal directed medical therapy [104–106]. Given the potential for SGLT2 inhibitors to mitigate the side effect of other medication classes, combination treatment with available guideline recommended treatments should be more achievable.

#### Acute HF

The optimal treatment approach for alleviating signs and symptoms of volume overload in patients with acute HF, particularly in the setting of diuretic resistance, remains unestablished. Urgent requirements for rapid diuresis have led to the emergence of a targeted multi-nephron segment approach, using rapid or sequential initiation of combined CA inhibitors, loop diuretics, thiazide diuretics, and MR antagonists [107, 108]. In a retrospective study of acute HF patients with severe diuretic resistance, high-intensity treatment using this multi-nephron segment approach resulted in greater diuresis and achievement of successful decongestion, compared to standard care [107]. Analogous the ADVOR trial, the use of background SGLT2 inhibitors was rare in the current study, and therefore the efficacy of dual proximal tubular natriuretic blockade with concurrent SGLT2 and CA inhibitors remains unknown. In addition, the order in which to introduce adjuncts to loop diuretics, and whether synergistic effects exist, remains largely unknown. However, it is notable that the diuretic response to CA inhibition in ADVOR was less than that observed with thiazide diuretics and SGLT2 inhibitors observed in the CLOROTIC and EMPAG-HF trials [48, 69]. Furthermore, it is also important to consider that the EMPULSE, ADVOR, and CLOROTIC trials have shown no effect of SGLT2 inhibition, CA inhibition, or thiazide diuretics on 30-day rehospitalization in patients with acute HF. Further prospective work is required to determine the efficacy of this intensive approach for achieving decongestion.

## SUMMARY

Contemporary adjunct proximal and distal diuretics have been shown to elicit clear clinical benefit in the setting of acute and chronic HF. SGLT2 inhibitors and MR antagonists both cause short-term increases in natriuresis and diuresis; however, these mechanisms may not represent the primary actions responsible for long-term cardioprotection in chronic HF patients. Similarly, short-term treatment with SGLT2 inhibitors, CA inhibitors, and thiazide diuretics increase natriuresis in the acute HF setting, however, implications on long-term outcomes remain unclear and warrant further investigation to elucidate optimal treatment regimes. Last, a multi-nephron segment approach, using agents that act on distinct segments of the nephron, can further augment diuresis to alleviate signs and symptoms of volume overload in the acute setting. Future trials are required to determine efficacy of the multi-nephron segment approach whereby mechanisms of multiple therapies converge on similar nephron segments.

## Data Availability

No new data were generated or analysed in support of this research.

## References

[1] Mullens W, Verbrugge FH, Nijst P et al. Renal sodium avidity in heart failure: from pathophysiology to treatment strategies. Eur Heart J 2017;38:1872–82. 10.1093/eurheartj/ehx035 28329085

[2] Felker GM, Ellison DH, Mullens W et al. Diuretic therapy for patients with heart failure: *JACC* state-of-the-art review. J Am Coll Cardiol 2020;75:1178–95. 10.1016/j.jacc.2019.12.059 32164892

[3] Selvaraj S, Claggett B, Pozzi A et al. Prognostic implications of congestion on physical examination among contemporary patients with heart failure and reduced ejection fraction. Circulation 2019;140:1369–79. 10.1161/CIRCULATIONAHA.119.039920 31510768

[4] Januzzi JL, Zannad F, Anker SD et al. Prognostic importance of NT-proBNP and effect of Empagliflozin in the EMPEROR-reduced trial. J Am Coll Cardiol 2021;78:1321–32. 10.1016/j.jacc.2021.07.046 34556318

[5] McDonagh TA, Metra M, Adamo M et al. 2021 ESC guidelines for the diagnosis and treatment of acute and chronic heart failure: developed by the Task Force for the diagnosis and treatment of acute and chronic heart failure of the European Society of Cardiology (ESC) with the special contribution of the Heart Failure Association (HFA) of the ESC. Eur Heart J 2021;42:3599–726.34447992

[6] Heidenreich PA, Bozkurt B, Aguilar D et al. 2022 AHA/ACC/HFSA Guideline for the management of heart failure: executive summary. J Am Coll Cardiol 2022;79:1757–80. 35379504 10.1016/j.jacc.2021.12.011

[7] Mullens W, Damman K, Harjola V-P et al. The use of diuretics in heart failure with congestion—A position statement from the Heart Failure Association of the European Society of Cardiology. Eur J Heart Fail 2019;21:137–55. 10.1002/ejhf.1369 30600580

[8] Kiernan MS, Stevens SR, Tang WHW et al. Determinants of diuretic responsiveness and associated outcomes during acute heart failure hospitalization: an analysis from the NHLBI heart failure network clinical trials. J Card Fail 2018;24:428–38. 10.1016/j.cardfail.2018.02.002 29482026 PMC6102061

[9] Neuberg GW, Miller AB, O'Connor CM et al. Diuretic resistance predicts mortality in patients with advanced heart failure. Am Heart J 2002;144:31–38. 10.1067/mhj.2002.123144 12094185

[10] Khan MS, Greene SJ, Hellkamp AS et al. Diuretic changes, health care resource utilization, and clinical outcomes for heart failure with reduced ejection fraction: from the change the management of patients with heart failure registry. Circulation: Heart Failure 2021;14:e008351. 34674536 10.1161/CIRCHEARTFAILURE.121.008351

[11] Chatur S, Vaduganathan M, Claggett B et al. Dapagliflozin and diuretic utilization in heart failure with mildly reduced or preserved ejection fraction: the DELIVER trial. Eur Heart J 2023;44:2930–43. 10.1093/eurheartj/ehad283 37220093 PMC10484057

[12] Butler J, Usman MS, Filippatos G et al. Safety and efficacy of Empagliflozin and diuretic use in patients with heart failure and preserved ejection fraction: a post hoc analysis of the EMPEROR-preserved trial. JAMA Cardiol 2023;8:640–9. 10.1001/jamacardio.2023.1090 37223933 PMC10209829

[13] Mullens W, Schulze PC, Westphal J et al. Great debate: in patients with decompensated heart failure, acetazolamide in addition to loop diuretics is the first choice. Eur Heart J 2023;44:2159–69. 10.1093/eurheartj/ehad266 37207453 PMC10290873

[14] Packer M, Anker SD, Butler J et al. Cardiovascular and renal outcomes with Empagliflozin in heart failure. N Engl J Med 2020;383:1413–24. 10.1056/NEJMoa2022190 32865377

[15] McMurray JJV, Solomon SD, Inzucchi SE et al. Dapagliflozin in patients with heart failure and reduced ejection fraction. N Engl J Med 2019;381:1995–2008. 10.1056/NEJMoa1911303 31535829

[16] Anker SD, Butler J, Filippatos G et al. Empagliflozin in heart failure with a preserved ejection fraction. N Engl J Med 2021;385:1451–61. 10.1056/NEJMoa2107038 34449189

[17] Solomon SD, McMurray JJV, Claggett B et al. Dapagliflozin in heart failure with mildly reduced or preserved ejection fraction. N Engl J Med 2022;387:1089–98. 10.1056/NEJMoa2206286 36027570

[18] Bhatt DL, Szarek M, Steg PG et al. Sotagliflozin in patients with diabetes and recent worsening heart failure. N Engl J Med 2021;384:117–28. 10.1056/NEJMoa2030183 33200892

[19] Nassif ME, Windsor SL, Borlaug BA et al. The SGLT2 inhibitor dapagliflozin in heart failure with preserved ejection fraction: a multicenter randomized trial. Nat Med 2021;27:1954–60. 10.1038/s41591-021-01536-x 34711976 PMC8604725

[20] Vestri S, Okamoto MM, de Freitas HS et al. Changes in sodium or glucose filtration rate modulate expression of glucose transporters in renal proximal tubular cells of rat. J Membr Biol 2001;182:105–12. 10.1007/s00232-001-0036-y 11447502

[21] Lytvyn Y, Bjornstad P, Udell JA et al. Sodium glucose cotransporter-2 inhibition in heart failure. Circulation 2017;136:1643–58. 10.1161/CIRCULATIONAHA.117.030012 29061576 PMC5846470

[22] Onishi A, Fu Y, Patel R et al. A role for tubular Na+/H+ exchanger NHE3 in the natriuretic effect of the SGLT2 inhibitor empagliflozin. Am J Physiol Renal Physiol 2020;319:F712–28. 10.1152/ajprenal.00264.2020 32893663 PMC7642886

[23] Cherney DZI, Perkins BA, Soleymanlou N et al. Renal hemodynamic effect of sodium-glucose cotransporter 2 inhibition in patients with type 1 diabetes mellitus. Circulation 2014;129:587–97. 10.1161/CIRCULATIONAHA.113.005081 24334175

[24] Liu H, Sridhar VS, Boulet J et al. Cardiorenal protection with SGLT2 inhibitors in patients with diabetes mellitus: from biomarkers to clinical outcomes in heart failure and diabetic kidney disease. Metabolism 2022;126:154918. 10.1016/j.metabol.2021.154918 34699838

[25] Hesp AC, Schaub JA, Prasad PV et al. The role of renal hypoxia in the pathogenesis of diabetic kidney disease: a promising target for newer renoprotective agents including SGLT2 inhibitors? Kidney Int 2020;98:579–89. 10.1016/j.kint.2020.02.041 32739206 PMC8397597

[26] Griffin M, Rao VS, Ivey-Miranda J et al. Empagliflozin in heart failure. Circulation 2020;142:1028–39. 10.1161/CIRCULATIONAHA.120.045691 32410463 PMC7521417

[27] Rao VS, Ivey-Miranda JB, Cox ZL et al. Empagliflozin in heart failure: regional nephron sodium handling effects. J Am Soc Nephrol 2023;35:189–201. 10.1681/ASN.0000000000000269 38073038 PMC10843196

[28] Zannad F, Ferreira JP, Butler J et al. Effect of empagliflozin on circulating proteomics in heart failure: mechanistic insights into the EMPEROR programme. Eur Heart J 2022;43:4991–5002. 10.1093/eurheartj/ehac495 36017745 PMC9769969

[29] Schork A, Saynisch J, Vosseler A et al. Effect of SGLT2 inhibitors on body composition, fluid status and renin–angiotensin–aldosterone system in type 2 diabetes: a prospective study using bioimpedance spectroscopy. Cardiovasc Diabetol 2019;18:1–12. 10.1186/s12933-019-0852-y 30953516 PMC6451223

[30] Masuda T, Muto S, Fukuda K et al. Osmotic diuresis by SGLT2 inhibition stimulates vasopressin-induced water reabsorption to maintain body fluid volume. Physiol Rep 2020;8:e14360. 10.14814/phy2.14360 31994353 PMC6987478

[31] Mordi NA, Mordi IR, Singh JS et al. Renal and cardiovascular effects of SGLT2 inhibition in combination with loop diuretics in patients with type 2 diabetes and chronic heart failure. Circulation 2020;142:1713–24. 10.1161/CIRCULATIONAHA.120.048739 32865004 PMC7594536

[32] Kolwelter J, Kannenkeril D, Linz P et al. The SGLT2 inhibitor empagliflozin reduces tissue sodium content in patients with chronic heart failure: results from a placebo-controlled randomised trial. Clin Res Cardiol 2023;112:134–44. 10.1007/s00392-022-02119-7 36289063 PMC9849317

[33] Jensen J, Omar M, Kistorp C et al. Effects of empagliflozin on estimated extracellular volume, estimated plasma volume, and measured glomerular filtration rate in patients with heart failure (Empire HF Renal): a prespecified substudy of a double-blind, randomised, placebo-controlled trial. Lancet Diabetes Endocrinol 2021;9:106–16. 33357505 10.1016/S2213-8587(20)30382-X

[34] Nakashima M, Miyoshi T, Ejiri K et al. Effects of luseogliflozin on estimated plasma volume in patients with heart failure with preserved ejection fraction. ESC Heart Fail 2022;9:712–20. 10.1002/ehf2.13683 35267246 PMC8787977

[35] Fujiki S, Tanaka A, Imai T et al. Body fluid regulation via chronic inhibition of sodium–glucose cotransporter-2 in patients with heart failure: a post hoc analysis of the CANDLE trial. Clin Res Cardiol 2023;112:87–97. 10.1007/s00392-022-02049-4 35729430

[36] Packer M, Wilcox CS, Testani JM. Critical analysis of the effects of SGLT2 inhibitors on renal tubular sodium, water and chloride homeostasis and their role in influencing heart failure outcomes. Circulation 2023;148:354–72. 10.1161/CIRCULATIONAHA.123.064346 37486998 PMC10358443

[37] Mayne KJ, Staplin N, Keane DF et al. Effects of Empagliflozin on fluid overload, weight and blood pressure in chronic kidney disease. J Am Soc Nephrol 2023;35:202–15. 10.1681/ASN.0000000000000271 38082486 PMC7615589

[38] Omar M, Jensen J, Frederiksen PH et al. Effect of Empagliflozin on hemodynamics in patients with heart failure and reduced ejection fraction. J Am Coll Cardiol 2020;76:2740–51. 10.1016/j.jacc.2020.10.005 33272368

[39] Nassif ME, Qintar M, Windsor SL et al. Empagliflozin effects on pulmonary artery pressure in patients with heart failure. Circulation 2021;143:1673–86. 10.1161/CIRCULATIONAHA.120.052503 33550815

[40] Fitchett D, Inzucchi SE, Zinman B et al. Mediators of the improvement in heart failure outcomes with empagliflozin in the EMPA-REG OUTCOME trial. ESC Heart Fail 2021;8:4517–27. 10.1002/ehf2.13615 34605192 PMC8712833

[41] Mazer CD, Hare GMT, Connelly PW et al. Effect of Empagliflozin on erythropoietin levels, iron stores, and red blood cell morphology in patients with type 2 diabetes mellitus and coronary artery disease. Circulation 2020;141:704–7. 10.1161/CIRCULATIONAHA.119.044235 31707794

[42] Vaduganathan M, Claggett BL, Jhund P et al. Time to clinical benefit of Dapagliflozin in patients with heart failure with mildly reduced or preserved ejection fraction: a prespecified secondary analysis of the DELIVER randomized clinical trial. JAMA Cardiol 2022;7:1259–63. 10.1001/jamacardio.2022.3750 36190011 PMC9531091

[43] Sridhar VS, Neuen BL, Fletcher RA et al. Kidney protection with canagliflozin: a combined analysis of the randomized CANVAS program and CREDENCE trials. Diabetes Obes Metab 2023;25:2331–9. 10.1111/dom.15112 37184050

[44] Packer M, Anker SD, Butler J et al. Empagliflozin in patients with heart failure, reduced ejection fraction, and volume overload: eMPEROR-reduced trial. J Am Coll Cardiol 2021;77:1381–92. 10.1016/j.jacc.2021.01.033 33736819

[45] Jackson AM, Dewan P, Anand IS et al. Dapagliflozin and diuretic use in patients with heart failure and reduced ejection fraction in DAPA-HF. Circulation 2020;142:1040–54. 10.1161/CIRCULATIONAHA.120.047077 32673497 PMC7664959

[46] Damman K, Beusekamp JC, Boorsma EM et al. Randomized, double-blind, placebo-controlled, multicentre pilot study on the effects of empagliflozin on clinical outcomes in patients with acute decompensated heart failure (EMPA-RESPONSE-AHF). Eur J Heart Fail 2020;22:713–22. 10.1002/ejhf.1713 31912605

[47] Boorsma EM, Beusekamp JC, ter Maaten JM et al. Effects of empagliflozin on renal sodium and glucose handling in patients with acute heart failure. Eur J Heart Fail 2021;23:68–78. 10.1002/ejhf.2066 33251643 PMC8048437

[48] Schulze PC, Bogoviku J, Westphal J et al. Effects of early Empagliflozin initiation on diuresis and kidney function in patients with acute decompensated heart failure (EMPAG-HF). Circulation 2022;146:289–98. 10.1161/CIRCULATIONAHA.122.059038 35766022

[49] Yeoh SE, Osmanska J, Petrie MC et al. Dapagliflozin vs. metolazone in heart failure resistant to loop diuretics. Eur Heart J 2023;44:2966–77. 10.1093/eurheartj/ehad341 37210742 PMC10424881

[50] Voors AA, Angermann CE, Teerlink JR et al. The SGLT2 inhibitor empagliflozin in patients hospitalized for acute heart failure: a multinational randomized trial. Nat Med 2022;28:568–74. 10.1038/s41591-021-01659-1 35228754 PMC8938265

[51] Friedberg CK, Taymor R, Minor JB et al. The use of Diamox, a carbonic anhydrase inhibitor, as an oral diuretic in patients with congestive heart failure. N Engl J Med 1953;248:883–9. 10.1056/NEJM195305212482102 13046634

[52] Hanley T, Platts MM. Acetazolamide (Diamox) in the treatment of congestive heart failure. Lancet North Am Ed 1956;267:357–9. 10.1016/S0140-6736(56)90105-2 13307929

[53] Verbrugge FH, Martens P, Ameloot K et al. Acetazolamide to increase natriuresis in congestive heart failure at high risk for diuretic resistance. Eur J Heart Fail 2019;21:1415–22. 10.1002/ejhf.1478 31074184

[54] Mullens W, Dauw J, Martens P et al. Acetazolamide in acute decompensated heart failure with volume overload. N Engl J Med 2022;387:1185–95. 10.1056/NEJMoa2203094 36027559

[55] Verbrugge FH, Martens P, Dauw J et al. Natriuretic response to acetazolamide in patients with acute heart failure and volume overload. J Am Coll Cardiol 2023;81:2013–24. 10.1016/j.jacc.2023.03.400 37197845

[56] Martens P, Verbrugge FH, Dauw J et al. Pre-treatment bicarbonate levels and decongestion by acetazolamide: the ADVOR trial. Eur Heart J 2023;44:1995–2005. 10.1093/eurheartj/ehad236 37138385

[57] Wilcox CS, Testani JM, Pitt B. Pathophysiology of diuretic resistance and its implications for the management of chronic heart failure. Hypertension 2020;76:1045–54. 10.1161/HYPERTENSIONAHA.120.15205 32829662 PMC10683075

[58] Martens P, Dauw J, Verbrugge FH et al. Decongestion with acetazolamide in acute decompensated heart failure across the spectrum of left ventricular ejection fraction: a prespecified analysis from the ADVOR trial. Circulation 2023;147:201–11. 10.1161/CIRCULATIONAHA.122.062486 36335479

[59] Rao VS, Planavsky N, Hanberg JS et al. Compensatory distal reabsorption drives diuretic resistance in Human heart failure. J Am Soc Nephrol 2017;28:3414. 10.1681/ASN.2016111178 28739647 PMC5661276

[60] Cox ZL, Hung R, Lenihan DJ et al. Diuretic strategies for loop Diuretic resistance in acute heart failure: the 3T trial. JACC: Heart Fail 2020;8:157–68. 31838029 10.1016/j.jchf.2019.09.012 PMC7058489

[61] Brisco-Bacik MA, ter Maaten JM, Houser SR et al. Outcomes associated with a strategy of adjuvant metolazone or high-dose loop diuretics in acute decompensated heart failure: a propensity analysis. J Am Heart Assoc 2018;7:e009149. 10.1161/JAHA.118.009149 30371181 PMC6222930

[62] Llàcer P, Núñez J, García M et al. Comparison of chlorthalidone and spironolactone as additional diuretic therapy in patients with acute heart failure and preserved ejection fraction. Eur Heart J Acute Cardiovasc Care 2022;11:350–5. 10.1093/ehjacc/zuac006 35167653

[63] Cisowska T, Pan IZ, Biskupiak J et al. Metolazone versus intravenous chlorothiazide for decompensated heart failure sequential nephron blockade: a retrospective cohort study. J Card Fail 2022;28:1367–71. 10.1016/j.cardfail.2022.05.011 35688407

[64] Sigurd B, Olesen KH, Wennevold A. The supra-additive natriuretic effect addition of bendroflumethiazide and bumetanide in congestive heart failure: permutation trial tests in patients in long-term treatment with bumetanide. Am Heart J 1975;89:163–70. 10.1016/0002-8703(75)90041-1 1090132

[65] Palazzuoli A, Ruocco G, Severino P et al. Effects of metolazone administration on congestion, diuretic response and renal function in patients with advanced heart failure. J Clin Med 2021;10:4207. 10.3390/jcm10184207 34575318 PMC8465476

[66] Dormans TPJ, Gerlag PGG. Combination of high-dose furosemide and hydrochlorothiazide in the treatment of refractory congestive heart failure. Eur Heart J 1996;17:1867–74. 10.1093/oxfordjournals.eurheartj.a014805 8960430

[67] Kiyingi A, Field MJ, Pawsey CC et al. Metolazone in treatment of severe refractory congestive cardiac failure. Lancet North Am Ed 1990;335:29–31. 10.1016/0140-6736(90)90148-X 1967337

[68] Channer KS, McLean KA, Lawson-Matthew P et al. Combination diuretic treatment in severe heart failure: a randomised controlled trial. Heart 1994;71:146–50. 10.1136/hrt.71.2.146 PMC483634 8130022

[69] Trulls JC, Morales-Rull JL, Casado J et al. Combining loop with thiazide diuretics for decompensated heart failure: the CLOROTIC trial. Eur Heart J 2023;44:411–21. 10.1093/eurheartj/ehac689 36423214

[70] Duncan LE, Liddle GW, Bartter FC et al. The effect of changes in body sodium on extracellular fluid volume and aldostrone and soidu excretion by normal and edematous men. J Clin Invest 1956;35:1299–305. 10.1172/JCI103385 13376723 PMC441708

[71] Agarwal R, Kolkhof P, Bakris G et al. Steroidal and non-steroidal mineralocorticoid receptor antagonists in cardiorenal medicine. Eur Heart J 2021;42:152–61. 10.1093/eurheartj/ehaa736 33099609 PMC7813624

[72] Kintscher U, Edelmann F. The non-steroidal mineralocorticoid receptor antagonist finerenone and heart failure with preserved ejection fraction. Cardiovasc Diabetol 2023;22:162. 10.1186/s12933-023-01899-0 37386461 PMC10311906

[73] Pitt B, Kober L, Ponikowski P et al. Safety and tolerability of the novel non-steroidal mineralocorticoid receptor antagonist BAY 94-8862 in patients with chronic heart failure and mild or moderate chronic kidney disease: a randomized, double-blind trial. Eur Heart J 2013;34:2453–63. 10.1093/eurheartj/eht187 23713082 PMC3743070

[74] Bakris GL, Agarwal R, Anker SD et al. Effect of finerenone on chronic kidney disease outcomes in type 2 diabetes. N Engl J Med 2020;383:2219–29. 10.1056/NEJMoa2025845 33264825

[75] Kobayashi M, Girerd N, Zannad F. When to use either spironolactone, eplerenone or finerenone in the spectrum of cardiorenal diseases. Nephrol Dial Transplant 2024. 10.1093/ndt/gfae004 38192033

[76] Pitt B, Zannad F, Remme WJ et al. The effect of spironolactone on morbidity and mortality in patients with severe heart failure. N Engl J Med 1999;341:709–17. 10.1056/NEJM199909023411001 10471456

[77] Zannad F, McMurray JJV, Krum H et al. Eplerenone in patients with systolic heart failure and mild symptoms. N Engl J Med 2011;364:11–21. 10.1056/NEJMoa1009492 21073363

[78] Pitt B, Pfeffer MA, Assmann SF et al. Spironolactone for heart failure with preserved ejection fraction. N Engl J Med 2014;370:1383–92. 10.1056/NEJMoa1313731 24716680

[79] Pfeffer MA, Claggett B, Assmann SF et al. Regional variation in patients and outcomes in the treatment of preserved cardiac function heart failure with an aldosterone antagonist (TOPCAT) trial. Circulation 2015;131:34–42. 10.1161/CIRCULATIONAHA.114.013255 25406305

[80] Filippatos G, Anker SD, Böhm M et al. A randomized controlled study of finerenone vs. eplerenone in patients with worsening chronic heart failure and diabetes mellitus and/or chronic kidney disease. Eur Heart J 2016;37:2105–14. 10.1093/eurheartj/ehw132 27130705 PMC4946749

[81] Farrelly RO, Howie RN, North JDK. Use of spironolactone and hydrochlorothiazide in treatment of oedema. Br Med J (Clin Res Ed) 1960;2:339–43. 10.1136/bmj.2.5195.339 PMC2097506 13821638

[82] Hensen J, Abraham WT, Dürr JA et al. Aldosterone in congestive heart failure: analysis of determinants and role in sodium retention. Am J Nephrol 1991;11:441–6. 10.1159/000168356 1840232

[83] van Vliet André A, Donker AJM, Nauta JJP et al. Spironolactone in congestive heart failure refractory to high-dose loop diuretic and low-dose angiotensin-converting enzyme inhibitor. Am J Cardiol 1993;71:A21–8. 10.1016/0002-9149(93)90241-4 8422000

[84] Rousseau MF, Gurné O, Duprez D et al. Beneficial neurohormonal profile of spironolactone in severe congestive heart failure: results from the RALES neurohormonal substudy. J Am Coll Cardiol 2002;40:1596–601. 10.1016/S0735-1097(02)02382-3 12427411

[85] Cleland JGF, Ferreira JP, Mariottoni B et al. The effect of spironolactone on cardiovascular function and markers of fibrosis in people at increased risk of developing heart failure: the heart ‘OMics’ in AGEing (HOMAGE) randomized clinical trial. Eur Heart J 2021;42:684–96. 10.1093/eurheartj/ehaa758 33215209 PMC7878013

[86] Bedrouni W, Sharma A, Pitt B et al. Timing of statistical benefit of mineralocorticoid receptor antagonists among patients with heart failure and post-myocardial infarction. Circ Heart Fail 2022;15:e009295. 35924555 10.1161/CIRCHEARTFAILURE.121.009295

[87] Kalogeropoulos AP, Thankachen J, Butler J et al. Diuretic and renal effects of spironolactone and heart failure hospitalizations: a TOPCAT Americas analysis. Eur J Heart Fail 2020;22:1600–10. 10.1002/ejhf.1917 32469156

[88] Anand IS, Claggett B, Liu J et al. Interaction between spironolactone and natriuretic peptides in patients with heart failure and preserved ejection fraction: from the TOPCAT trial. JACC: Heart Fail 2017;5:241–52. 28359411 10.1016/j.jchf.2016.11.015

[89] Butler J, Anstrom KJ, Felker GM et al. Efficacy and safety of spironolactone in acute heart failure: the ATHENA-HF randomized clinical trial. JAMA Cardiol 2017;2:950–8. 10.1001/jamacardio.2017.2198 28700781 PMC5675712

[90] Ferreira JP, Girerd N, Zannad F. Interpretation of the ATHENA trial—caveats and future directions. JAMA Cardiol 2018;3:89–90. 10.1001/jamacardio.2017.4370 29214308

[91] Goldsmith SR, Gheorghiade M. Vasopressin antagonism in heart failure. J Am Coll Cardiol 2005;46:1785–91. 10.1016/j.jacc.2005.02.095 16286160

[92] Maisel A, Xue Y, Shah K et al. Increased 90-day mortality in patients with acute heart failure with elevated copeptin. Circ Heart Fail 2011;4:613–20. 21765124 10.1161/CIRCHEARTFAILURE.110.960096

[93] Gheorghiade M, Niazi I, Ouyang J et al. Vasopressin V2-receptor blockade with Tolvaptan in patients with chronic heart failure. Circulation 2003;107:2690–6. 10.1161/01.CIR.0000070422.41439.04 12742979

[94] Gheorghiade M, Gattis WA, O'Connor CM et al. Effects of Tolvaptan, a vasopressin antagonist, in patients hospitalized with worsening heart failure. A randomized controlled trial. JAMA 2004;291:1963–71. 10.1001/jama.291.16.1963 15113814

[95] Konstam MA, Gheorghiade M, Burnett JC et al. Effects of oral Tolvaptan in patients hospitalized for worsening heart failure: the EVEREST outcome trial. JAMA 2007;297:1319–31. 10.1001/jama.297.12.1319 17384437

[96] Felker GM, Mentz RJ, Cole RT et al. Efficacy and safety of Tolvaptan in patients hospitalized with acute heart failure. J Am Coll Cardiol 2017;69:1399–406. 10.1016/j.jacc.2016.09.004 27654854

[97] Konstam MA, Kiernan M, Chandler A et al. Short-term effects of Tolvaptan in patients with acute heart failure and volume overload. J Am Coll Cardiol 2017;69:1409–19. 10.1016/j.jacc.2016.12.035 28302292

[98] Schrier RW, Gross P, Gheorghiade M et al. Tolvaptan, a selective oral Vasopressin V2-receptor antagonist, for hyponatremia. N Engl J Med 2006;355:2099–112. 10.1056/NEJMoa065181 17105757

[99] Tromp J, Ouwerkerk W, van Veldhuisen DJ et al. A systematic review and network meta-analysis of pharmacological treatment of heart failure with reduced ejection fraction. JACC: Heart Fail 2022;10:73–84. 34895860 10.1016/j.jchf.2021.09.004

[100] Zafeiropoulos S, Farmakis IT, Milioglou I et al. Pharmacological treatments in heart failure with mildly reduced and preserved ejection fraction: systematic review and network meta-analysis. JACC: Heart Fail 2023. Available from: https://www.sciencedirect.com/science/article/pii/S2213177923004109 10.1016/j.jchf.2023.07.014 37656079

[101] Packer M, McMurray JJV. Rapid evidence-based sequencing of foundational drugs for heart failure and a reduced ejection fraction. Eur J Heart Fail 2021;23:882–94. 10.1002/ejhf.2149 33704874 PMC8360176

[102] Greene SJ, Butler J, Fonarow GC. Simultaneous or rapid sequence initiation of quadruple medical therapy for heart failure—optimizing therapy with the need for speed. JAMA Cardiol 2021;6:743–4. 10.1001/jamacardio.2021.0496 33787823

[103] Mebazaa A, Davison B, Chioncel O et al. Safety, tolerability and efficacy of up-titration of guideline-directed medical therapies for acute heart failure (STRONG-HF): a multinational, open-label, randomised, trial. Lancet 2022;400:1938–52. 10.1016/S0140-6736(22)02076-1 36356631

[104] Ferreira JP, Zannad F, Pocock SJ et al. Interplay of mineralocorticoid receptor antagonists and empagliflozin in heart failure: eMPEROR-reduced. J Am Coll Cardiol 2021;77:1397–407. 10.1016/j.jacc.2021.01.044 33736821

[105] Butler J, Anker SD, Lund LH et al. Patiromer for the management of hyperkalemia in heart failure with reduced ejection fraction: the DIAMOND trial. Eur Heart J 2022;43:4362–73. 10.1093/eurheartj/ehac401 35900838 PMC9622299

[106] Kristensen SL, Docherty KF, Jhund PS et al. Dapagliflozin reduces the risk of hyperkalaemia in patients with heart failure and reduced ejection fraction: a secondary analysis DAPA-HF. Eur Heart J 2020;41:ehaa946.0939. 10.1093/ehjci/ehaa946.0939

[107] Cox ZL, Sarrell BA, Cella MK et al. Multinephron segment diuretic therapy to overcome diuretic resistance in acute heart failure: a single-center experience. J Card Fail 2022;28:21–31. 10.1016/j.cardfail.2021.07.016 34403831

[108] Kazory A. Combination diuretic therapy to counter renal sodium avidity in acute heart failure: trials and tribulations. Clin J Am Soc Nephrol 2023;18:1372. 10.2215/CJN.0000000000000188 37102974 PMC10578637

